# Student engagement and wellbeing over time at a higher education institution

**DOI:** 10.1371/journal.pone.0225770

**Published:** 2019-11-27

**Authors:** Chris A. Boulton, Emily Hughes, Carmel Kent, Joanne R. Smith, Hywel T. P. Williams

**Affiliations:** 1 Computer Science, University of Exeter, Exeter, United Kingdom; 2 School of Psychology, University of Exeter, Exeter, United Kingdom; University of Reading, UNITED KINGDOM

## Abstract

Student engagement is an important factor for learning outcomes in higher education. Engagement with learning at campus-based higher education institutions is difficult to quantify due to the variety of forms that engagement might take (e.g. lecture attendance, self-study, usage of online/digital systems). Meanwhile, there are increasing concerns about student wellbeing within higher education, but the relationship between engagement and wellbeing is not well understood. Here we analyse results from a longitudinal survey of undergraduate students at a campus-based university in the UK, aiming to understand how engagement and wellbeing vary dynamically during an academic term. The survey included multiple dimensions of student engagement and wellbeing, with a deliberate focus on self-report measures to capture students’ subjective experience. The results show a wide range of engagement with different systems and study activities, giving a broad view of student learning behaviour over time. Engagement and wellbeing vary during the term, with clear behavioural changes caused by assessments. Results indicate a positive interaction between engagement and happiness, with an unexpected negative relationship between engagement and academic outcomes. This study provides important insights into subjective aspects of the student experience and provides a contrast to the increasing focus on analysing educational processes using digital records.

## Introduction

Engagement with learning is believed to be an important factor in student success in higher education. Engagement has been defined in different ways in the literature [1], but is considered here to refer to the active commitment and purposeful effort expended by students towards all aspects of their learning, including both formal and informal activities [2]. Student engagement has been shown to be related to success in both online learning [3–5] and more traditional campus-based higher education settings [6–8]. However, engagement can be difficult to measure. In most studies of online-only education (e.g. [9–13]), student engagement is measured from the interactions a student has within a virtual learning environment (VLE). This may be a reasonable approach for digital-only contexts where a large proportion of learning activities occur through this channel. In contrast, in a traditional, face-to-face learning, university environment, VLE usage only captures one dimension of student learning activity and full engagement with learning is much harder to measure. The numerous and varied interactions students have with their learning programmes, including lectures, seminars, peer group discussions and *ad hoc* interactions with teaching staff, as well as other aspects of campus life such as participation in sports and student societies, are harder to record, requiring innovative methods for their capture [14, 15].

Exploration of the relationship between student engagement and success raises the important question of how “success” is defined. Most obviously, success relates to academic performance, such as final grades (e.g. [6–8, 16]), but success is also often discussed in terms of retention and completion of a course of learning (e.g. [7, 10, 13, 17–19]). It is important to consider that students may have different motivations for attending university, including, for example, social or sporting aims alongside conventional academic goals. Thus, in seeking to link engagement to success, there is value in adopting a more holistic view of student motivations and appropriate measures of outcomes. Furthermore, it is important to note that engagement and success, however measured, are dynamic and should be expected to vary within and between individuals over the duration of academic study.

There is increasing interest in learning analytics [20–25], which may use either static attributes of students (e.g. demographics, socioeconomic indicators, previous attainment) or dynamic attributes based on digital traces of learning behaviour to understand many aspects of the student experience, including student engagement. Traditionally, such studies have primarily made use of “found” data from institutional databases and “by-product” data from digital learning platforms. This kind of data, which is not collected for the purpose of pedagogical research, has limitations. The records that are collected institutionally tend to relate to either the administration of higher education (e.g., demographic data, recruitment/retention statistics) or to the core components of academic performance (e.g., grades, progression, completion). Data collected as the by-product of student learning activities on digital platforms such as VLEs (e.g. [8–10] only offers a partial view of a complex whole. For example, previous work that examined the relationship between academic performance and engagement at a traditional University found that VLE usage alone is a relatively poor predictor of academic performance in this context [8], while another study showed that VLE usage was a useful predictor of outcomes for online learning but not significant for face-to-face learning [9].

Dispositional learning analytics (see [26]), on the other hand, seeks to combine digital trace data (e.g., those generated by engagement in online learning activities) with learner data (e.g., dispositions, attitudes, and values assessed via self-report surveys). By doing so, recent research has found that learning dispositions (e.g., motivation, emotion, self-regulation) strongly and dynamically influence engagement and academic performance over time (e.g., [27–29]). In addition, this research suggests that the predictive value added by consideration of learner data might be time-dependent: learner data seems to play a critical role up until the point that feedback from assessment or online activities becomes available. This raises the possibility that whether incorporating learner dispositions into learning analytics models is useful depends on learning context (i.e., online only versus campus-based institutions).

Another limitation of learning analytics based solely on digital traces, is that these sources often cannot capture subjective aspects of student life, such as wellbeing and satisfaction, which are rarely routinely measured. Relationships between student engagement and wellbeing, or between wellbeing and success, have consequently been less well studied for higher education than that between engagement and success (but see [30, 31]). One project that has moved beyond by-product data and used deliberate collection of digital records to measure student behaviour and wellbeing is the StudentLife study at Dartmouth College in the USA [14]. This project supplied mobile phones to student participants in a term-long study that attempted to capture a multi-dimensional and longitudinal view of student behaviour. Findings used aspects of student life that had previously been inaccessible to researchers, including social interactions and physical activity patterns, to predict academic performance [16] and also to diagnose wellbeing issues [14, 32]. While the StudentLife study showed that deliberate data collection using digital methods can access important aspects of the subjective student experience, it does not address the difficulty of doing so using the kinds of by-product digital records and institutional data that are routinely collected and used as input into learning analytics.

The importance of student wellbeing for academic outcomes, and the relationships between wellbeing and engagement, remain open research questions for higher education. Wellbeing is a loosely defined concept that may include a number of different dimensions, including satisfaction, positive affect (e.g. enjoyment, gratitude, contentment) and negative affect (e.g. anger, sadness, worry) [33, 34]. Many studies have explored the relationship between wellbeing and academic performance, commonly finding a positive association, e.g. in US college undergraduates [35, 36] and among high school students [37]. The relationship between engagement and wellbeing is less well studied in higher education, but a positive association has been found in other working environments [34]. A recent government report on student mental health and wellbeing in UK universities found increasing incidence of mental illness, mental distress and low wellbeing [38]. The same study found that these negative wellbeing factors had a substantial harmful impact on student performance and course completion; by extension, students with positive wellbeing are likely to perform better and complete their studies. Another study by the UK Higher Education Academy focused on methods for promoting wellbeing in higher education, as well as identifying several pedagogical benefits [39].

Here we report on a longitudinal survey of student learning behaviours at a traditional campus-based university in the United Kingdom. Our survey was designed to capture multiple dimensions of student engagement and wellbeing over time, deliberately using self-report to look beyond digital traces and institutional records. An initial questionnaire included questions to characterise individual students on different dimensions including learning style and motivations for study. Subsequent waves captured student learning behaviours and engagement with a wide variety of learning systems (both offline and online) and activities, as well as their subjective feelings of satisfaction and wellbeing. The survey ran in 10 waves spanning a teaching semester, vacation and exam period, allowing observation of changes over time.

This study aims to complement the growing body of work that uses digital trace data to measure engagement, with a more subjective offline approach that captures a fuller representation of the student experience. Our research goals are to understand how engagement and wellbeing vary over time, as well as to determine a multidimensional view of student learning behaviours and patterns. Addressing these questions will make an important contribution to the academic study of student engagement and will help to identify other learning dispositions (e.g., engagement) that might be of value to combine with digital trace data in learning analytic models. Findings may also offer instrumental benefit by helping to guide institutional decision-making around interventions and student support.

## Methods

### Survey

The cohort for the survey consisted of 1st year and 2nd year undergraduate students at a research-intensive campus-based university in the United Kingdom. Students were invited to participate via emails containing a link to survey registration. In addition, recruitment booths were set up at the university’s main campus and researchers approached students to invite them to participate. Students were incentivised by entry into a prize draw to win gift vouchers for a well-known online retailer, with 10 prizes available in each wave. There were 10 waves in all. To incentivise continued participation, there was an additional final prize draw with larger prizes available to students who had completed 80% of surveys. Every participant explicitly gave their consent to their data being analysed for research purposes.

The survey ran from February to June 2017. Of the 10 waves, Waves 1–7 were released weekly during the Spring term, followed by a break for the Easter vacation period. Waves 8–10 were released fortnightly during the Summer term, which at this institution was mostly taken up with revision and examinations. Responses were received asynchronously, so although the survey was released in waves, we analyse the data over a continuous time interval spanning 19 weeks.

Our longitudinal survey consisted of a series of questions that students completed in every wave. To measure engagement with learning, we asked respondents to report their participation in each of 17 different learning activities (see Table 1), measured as the number of days in the past 7 days they had performed that activity. These activities were selected to represent the variety of online and offline activities, as well as social and academic activities, available to students at the university. To give context, we also asked respondents to report whether they had an assessment due in the past 7 days.

**Table 1:** Learning activities included in the survey.

Learning activity	Description
Work with friends	Work with friends on coursework or revision.
Interact with lecturer	Talk with a lecturer outside of a scheduled teaching session to aid their learning.
Use info app	Using the mobile phone app where students can access timetable, module results, and get emails etc.
Use VLE	Using the university’s virtual learning environment.
Attend teaching session	Attend a scheduled lecture, seminar or tutorial.
Access library	Access library resources, either physical books or online.
Use sports facilities	Go to the on-campus gym or play sports (outside of a club).
Use career services	Attend events created by the university to aid in students’ future employability.
Use SU facilities	Made use of student union facilities such as the student-run advice helpline.
Use retail facilities	Buy things on campus (a proxy for a student being on campus).
Catering facilities	Specifically buying food on campus (also includes accommodation food).
Use social media for learning	Finding information needed for learning on social media sites.
Use the internet for learning	Otherwise using the internet for learning.
View past exams	Revising for exams by looking at past exam papers provided by the university.
Go to clubs or societies	Attend sports clubs or societies outside of learning.
Talk to year rep.	Talk to an elected student representative who liaises with the university concerning problems
Accessed lecture recordings	Viewed recorded lectures or other teaching sessions for revision or for catching up on missed information.

Effort over the preceding week was assessed with two items assessed on a 5-point Likert scale (specifically, “How engaged were you with your studies?”; “How much effort did you put into your studies?”, 1 = not at all, 5 = very much). The mean response from each student was used to form a reliable scale (Pearson’s *r* = 0.78, *p* < .001). Well-being over the last week was assessed with four items that asked about happiness in general (e.g., “How happy did you feel about your life in general?”) and in relation to their programme of study (e.g., “How well do you feel you are doing in your course?”, 1 = not at all, 5 = very much). Responses were averaged to form a reliable scale (Cronbach’s α = 0.69).

In addition to the longitudinal survey questions, we also asked further questions in Wave 1 to determine their self-reported learning engagement style and motivation for attending university.

Engagement with learning was assessed with 10 items adapted from the Student Engagement in Schools Questionnaire (SESQ; [40]). Participants indicated the extent of their agreement with the statements on a 5-point Likert scale (1 = strongly disagree, 5 = strongly agree). Principal components analysis with varimax rotation extracted two factors, accounting for 53% of the variance. The first factor was characterised by the items assessing cognitive engagement (e.g., “When I study, I try to understand the material better by relating it to things I already know”), and items were averaged to form a cognitive engagement scale (α = 0.73). The second factor was characterised by the items assessing behavioural engagement (e.g., “In my modules, I work as hard as I can”), and items were averaged to form a behavioural engagement scale (α = 0.75).

Participants indicated their agreement with six different reasons for attending university (1 = not at all, 5 = very much). Principal components analysis with varimax rotation extracted two factors, accounting for 57% of the variance. The first factor was characterised by the items assessing social motivations (e.g., “To socialise with friends”), and items were averaged to form a social motivations scale (α = 0.62). The second factor was characterised by the items assessing academic motivations (e.g., “To get good grades”), and items were averaged to form an academic motivations scale (α = 0.48). The original survey is shown in Supplementary Information (S1 File).

The survey and following analysis were undertaken in accordance with the guidelines of the British Psychological Society. All participants provided informed consent prior to participation and were free to withdraw at any time without penalty. The survey and analysis received ethical approval from the University of Exeter Psychology Ethics Committee prior to commencement of data collection.

### Analysis

Our analysis is based on both static and dynamic variables from the survey responses for each student. Static variables include the motivation and engagement style measurements that were calculated from Wave 1. An additional static variable was also used to measure student academic performance across the term in which the survey was conducted, using grade data from the university database; for this metric, a student grade variable was calculated as their credit-weighted average grade from all the modules they took during the term in which the survey was conducted. Dynamic variables include the engagement and wellbeing measurements recorded in every wave. To allow comparison between static variables and dynamic variables, we take the mean value for the dynamic variable (e.g., the mean number of days per week that a student participated in a learning activity, or their mean effort scale score). Correlations between variables are measured using the Pearson correlation coefficient and measure correlations between both the static and dynamic variables. In both cases, all data is used in the correlation measurement, such that there is one record per student who answered in Wave 1, and all the responses are used to calculate the correlation between the dynamic variables.

Dynamic variables were used to analyse trends in behaviour over time, such as trends in engagement and wellbeing. To allow analysis of trends across the whole cohort, we created time series for engagement and wellbeing variables using a moving average across all responses with a 7-day window size. To ensure robustness, we made sure there were at least 10 responses in each window for which a mean was calculated. Since counts were lower during vacation and examination periods, we restricted our trend analysis to term-time only. Trends in these time series were calculated using the Kendall rank correlation coefficient, which counts the proportion of concordant pairs (both x_i_ >x_j_ and y_i_>y_j_ or x_i_<x_j_ and y_i_<y_j_). Using time as one of the variables, this gives a measure of tendency in the range [–1,1], with a score of -1 if the time series is always decreasing, a score of +1 if the time series is always increasing, and a score of 0 if there is no overall trend.

Our analysis involved looking for differences in behaviour between sub-populations within our respondent cohort (e.g. splitting the cohort into those who did or did not have an assessment due each week). We present differences in the mean values between the two distributions and then use a Mann-Whitney U-test to determine if the distributions are significantly different. We use these non-parametric tests since the distributions of values are typically non-normal and vary in shape between different variables. We also have a small sample size once the distributions have been split. However, we still present the difference in mean values, rather than the difference in median values, since the discrete nature of our data (e.g., integer values in range 0–7, which for some variables have an inter-quartile range of 0 to 1) means that medians are sometimes too coarse-grained to show differences even where the distributions are significantly different.

## Results

### Survey response

Overall, we had responses from 175 unique students, 174 of which answered the Wave 1 survey including questions to determine engagement style and motivations. We had 1050 responses overall, giving an average of exactly 6 responses per student.

**Fig 1:**
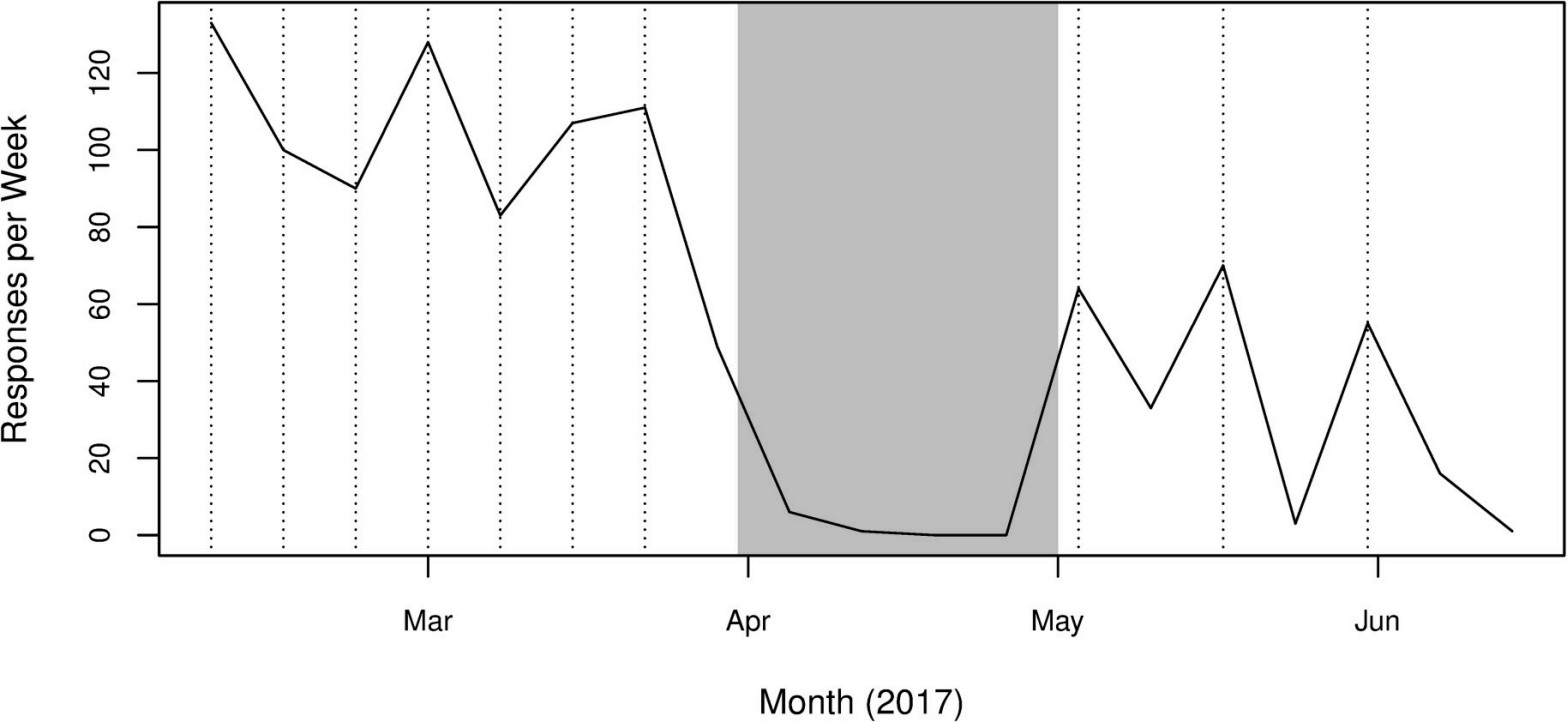
Weekly counts of survey responses over time. Grey shaded region refers to the Easter break between semesters. Spring Term is to the left of the grey region, Summer Term to the right. Vertical dotted lines indicate the weeks in which a survey email was sent and a responder lottery was held to incentivise participation. Note that students could answer a survey wave in the following week, hence a lower amount of first-week responses is observed when compared to the 174 students that answered the first wave of the survey.

Fig 1 shows the number of responses received over time during the 19-week period that the survey was active. There is an expected decline in the number of responses over time as participants lose interest or for other reasons drop out of the cohort. Despite this, we still have a reasonably steady and high response rate during the Spring term (left of the grey shaded area). There is a significant drop off in survey participation during the Easter break (grey shaded area), before the response rate recovers during the Summer term, although not to the levels seen previously (right of great shaded area). The Summer term in our survey is dominated by revision and exams, which suggests we might see different student behaviour.

Table 2 shows some demographics of our survey respondents (n = 175), compared to the entire student population (n = 15646). We find that our survey respondents are slightly biased towards being female and in their first year of study. The students who took the survey also have slightly higher marks than the student population. The number of students in the Life and Environmental Sciences college is greater than expected, with less representation of students from the Social Sciences and International Studies college and the Medical School. The low numbers from the Medical School reflect the fact that this School is based on a different campus to where physical recruitment of participants occurred.

**Table 2:** Demographic data for survey respondents and the student population for the 2016/17 academic year.

			Survey (%)		Student Population (%)	
Gender		Female	66		54	
		Male	34		46	
Year		First	49	67	31	63
		Second	27	64	31	63
Proportion	Average Grade	Third	21	70	31	66
		Other	3	71	7	68
College		Business School	11		18	
		Engineering, Mathematics and Physical Sciences	14		14	
		Medical School	1		7	
		Humanities	18		26	
		Social Sciences and International Studies	1		17	
		Life and Environmental Sciences	51		16	
		Other	4		2	

### Respondent characteristics

**Fig 2:**
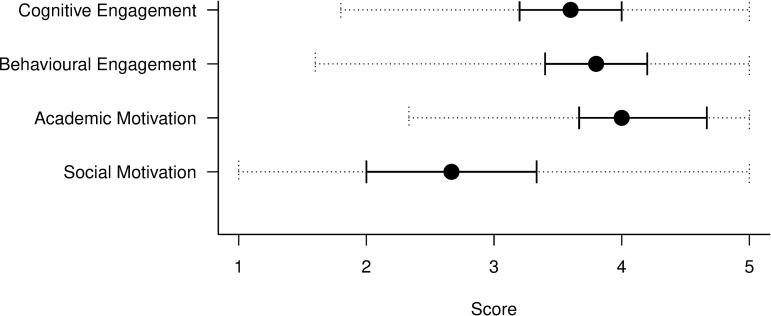
Distributions of scores of cognitive and behavioural engagement, and academic and social motivation from the Wave 1 survey responses. Students were asked a one-time set of questions to determine their engagement type and motivations (see Methods) and as such this is a static measurement. Dotted lines show the minimum and maximum scores, solid lines show the interquartile range, and points show the medians.

The Wave 1 survey included one-time questions intended to allow construction of engagement style and motivation scores for each individual student (see Methods). The distributions of these scores are shown in Fig 2. Due to the nature of these measurements, and the fact that they are only measured once, they make up part of our ‘static’ data and can be thought of as measuring a student’s underlying dispositions. They suggest that generally students reported slightly higher levels of behavioural engagement than cognitive engagement, although there was a bigger spread in behavioural engagement scores. Most of the students who responded to our survey reported higher academic motivation than social motivation for attending university.

### Relationships among student characteristics, average engagement and performance

**Fig 3:**
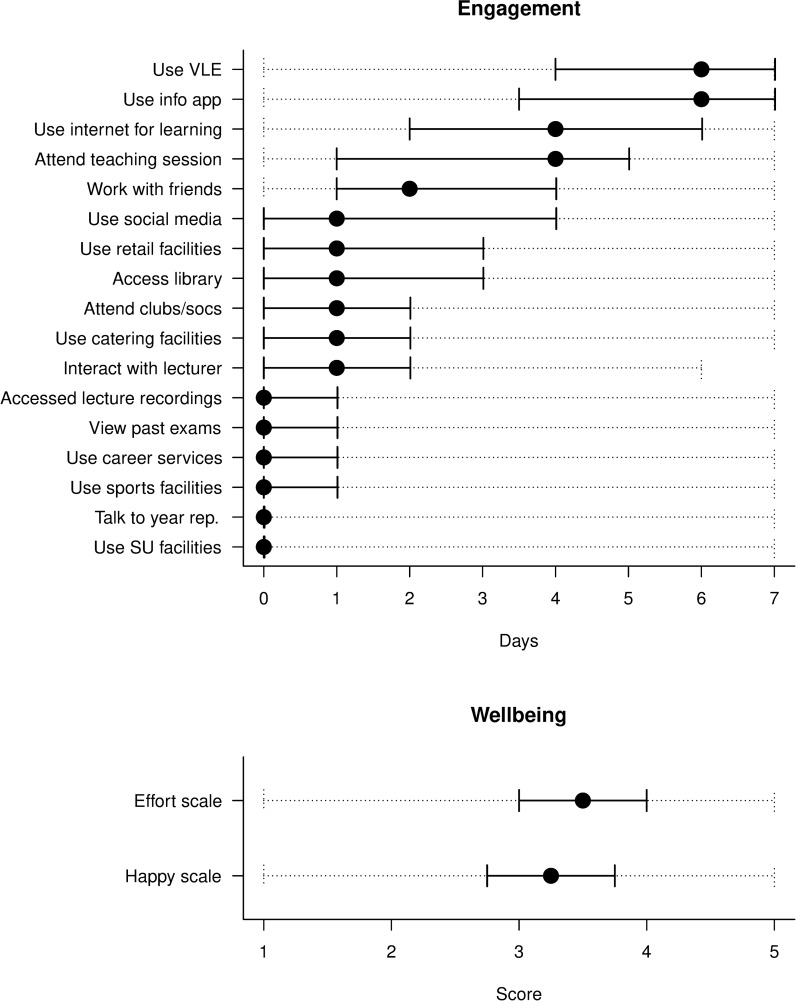
Distributions of engagement with different learning activities (days-per-week) and wellbeing (5-point scale from low to high) (see Methods). The underlying survey questions were asked in all waves and as such these are dynamic variables. Plot shows minimum and maximum scores (dotted lines), the interquartile range (solid lines) and median values (points). For this analysis all student responses were pooled.

Fig 3 shows the distributions of values from the longitudinal survey questions used to measure dynamic variables related to engagement with different learning activities and levels of student wellbeing. The plots show all responses from all students aggregated together, with the various learning activities ordered according to their mean usage level. The distributions suggest that activities that are most directly associated with learning (e.g. using the VLE, using the info app, using the Internet for learning, attending a teaching session) are used much more frequently than those that are not (e.g. using sports facilities, talking to a year representative, using SU facilities). This is consistent with the finding above that most students in the sample had stronger academic than social motivations for attending university. Distributions of scores on the “effort” and “happy” scales derived from the wellbeing questions asked each week (see Methods) show that both metrics have a broad absolute range but a relatively narrow interquartile range. These metrics cannot be usefully compared.

Next, we related the various static variables to each other and to the mean values for the various dynamic variables for each student in our cohort. Table 3 shows (Spearman’s) correlations between static variables across the cohort for: engagement style, motivation, grades, wellbeing, and engagement levels. Statistical significance is indicated in Table 3; henceforth we only discuss correlations with statistical significance at level *p*<0.05, unless stated explicitly. For the dynamic variables, we use the mean reported level across all responses for each student. Grades are analysed using the average credit-weighted module grade from the term in which the survey was carried out (see Methods).

**Table 3:** Static correlations in the survey.

	2	3	4	5	6	7	8	9	10	11	12	13	14	15	16	17	18	19	20	21	22	23	24*
**1. Cog. Eng.**	0.36*	**0.12**	**-0.12**	**0.02**	**0.07**	0.30*	**0.05**	0.32*	**0.03**	**-0.00**	**-0.13**	0.28*	**-0.08**	**0.09**	**0.15**	**0.07**	**0.05**	0.18*	0.24*	**-0.09**	**-0.03**	**0.01**	**-0.16**
**2. Behav. Eng.**		0.29*	**-0.22#**	0.24*	0.55*	**0.26**	**0.06**	**0.07**	**-0.06**	**0.02**	**-0.16#**	**0.08**	**-0.08**	**0.01**	**-0.04**	**-0.07**	**-0.07**	**0.01**	**0.13**	**0.02**	**-0.05**	**-0.01**	**-0.17#**
**3. Acad. Motiv.**			**0.14**	**0.13**	0.28*	0.29*	**0.09**	**0.05**	0.22*	0.23*	**0.12**	**0.03**	**0.11**	**0.06**	**-0.00**	**0.08**	**0.04**	**0.11**	**0.15**	**0.10**	**0.06**	**-0.07**	**0.09**
**4. Soc. Motiv.**				**-0.25#**	**0.09**	**0.21**	0.19*	**-0.06**	**0.07**	**0.10**	0.26*	**0.05**	0.46*	**-0.03**	**0.04**	0.20*	0.23*	0.21*	**0.10**	**0.05**	0.36*	**-0.01**	**0.02**
**5. Grades**					**-0.02**	**0.23**	**-0.03**	**-0.01**	**0.04**	**-0.06**	**-0.17#**	**-0.13**	**-0.04**	**-0.03**	**-0.11**	**-0.22#**	**-0.32#**	**-0.13**	**-0.13**	**0.01**	**-0.14**	**-0.07**	**0.14**
**6. Effort Scale**						0.30*	**0.11**	**0.03**	**0.11**	0.27*	**0.04**	0.31*	**0.00**	0.30*	**0.20**	0.27*	**0.24**	0.36*	0.50*	**0.05**	**0.21**	**0.09**	**0.06**
**7. Happy Scale**							**0.17**	**0.17**	**0.10**	**-0.04**	**0.06**	**0.12**	**0.06**	**0.07**	0.28*	**0.11**	**0.18**	**0.21**	**-0.02**	**-0.11**	0.36*	**-0.09**	**0.08**
**8. Friends**								0.24*	**-0.00**	0.24*	0.17*	0.20*	**0.14**	**0.03**	0.19*	0.44*	0.40*	0.31*	0.30*	0.37*	0.23*	0.16*	**0.02**
**9. Lecturer**									**0.12**	**0.05**	**-0.01**	0.29*	**-0.08**	**0.08**	0.26*	0.20*	**0.13**	**0.14**	0.17*	**0.03**	**0.05**	0.17*	**-0.03**
**10. Info App**										0.**0	0.33*	0.17*	**0.05**	**0.13**	**0.03**	**0.07**	**0.07**	0.24*	0.22*	0.20*	**0.04**	**-0.02**	0.20*
**11. VLE**											0.36*	0.31*	**0.11**	**0.06**	**0.03**	0.19*	0.15*	0.31*	0.46*	0.30*	0.16*	**0.10**	**0.12**
**12. Session**												**0.08**	0.24*	**-0.07**	**0.07**	0.30*	0.28*	0.16*	**0.11**	**0.09**	0.30*	**-0.05**	**0.03**
**13. Library**													**0.14**	0.20*	0.25*	0.33*	0.29*	0.26*	0.36*	**0.10**	**0.14**	**0.02**	**-0.09**
**14. Sports**														**0.01**	**0.04**	**0.13**	0.16*	**0.11**	**0.08**	**0.13**	0.31*	**0.04**	**0.02**
**15. Career**															0.30*	**0.04**	**0.07**	0.19*	**0.06**	**0.08**	**0.11**	**0.12**	**0.04**
**16. SU**																0.32*	0.30*	0.30*	**0.10**	**0.13**	0.31*	**0.04**	0.16*
**17. Retail**																	0.77*	0.27*	0.29*	**0.13**	0.35*	**0.01**	**0.02**
**18. Catering**																		0.30*	0.33*	0.17*	0.37*	**0.05**	**0.01**
**19. Soc. Med**																			0.45*	0.18*	0.24*	**0.09**	**-0.07**
**20. Internet**																				0.25*	0.16*	**0.03**	**-0.11**
**21. P. Exams**																					0.21*	0.19*	0.27*
**22. Socs**																						**0.07**	**0.06**
**23. Year Rep**																							**0.09**

Column 24 refers to ‘Lecture Recordings’.

Red boxes (*) refer to significantly (*p* < 0.05) positive correlations and blue (#) to significantly negative.

We find relatively strong positive correlation (ρ = 0.36) between levels of the two engagement styles (behavioural and cognitive). Behavioural engagement is correlated positively with academic motivation for attending university (ρ = 0.15) but correlated negatively with social motivation (ρ = -0.22). Behavioural engagement is very strongly positively correlated with effort (ρ = 0.55) and positively correlated with grades (ρ = 0.24). Cognitive engagement, on the other hand, is not correlated with grades (ρ = 0.02) but is positively correlated with happiness (ρ = 0.30). Cognitive engagement is also often positively correlated with participation in the various learning activities, with several positive correlations: seeing a lecturer (ρ = 0.32); going to the library (ρ = 0.28); using social media for learning (ρ = 0.18); and using the Internet for learning (ρ = 0.24). Cognitive engagement is negatively correlated with viewing lecture recordings (ρ = -0.16). Interestingly, behavioural engagement was typically uncorrelated with participation in learning activities except negatively with attending scheduled teaching sessions (ρ = -0.16); and viewing lecture recordings (ρ = -0.17).

The two types of motivation (academic and social) are not significantly correlated with each other (ρ = 0.14), but social motivation is correlated negatively with grades (ρ = -0.25). Academic motivation is significantly correlated with wellbeing scales for both effort (ρ = 0.28) and happiness (ρ = 0.29), whereas social motivation is not. Regarding participation in learning activities, the pattern of correlations makes intuitive sense. Academic motivation is weakly positively correlated with two academic activities: info app usage (ρ = 0.22); and VLE usage (ρ = 0.23). Social motivation is positively correlated with one core academic activity, attending a teaching session (ρ = 0.26), but is also positively correlated with several activities that are less directly academic and have a social aspect: working with friends (ρ = 0.19), using sports facilities (ρ = 0.46), using retail facilities (ρ = 0.23), using catering facilities (ρ = 0.23), using social media for learning (ρ = 0.21), and attending clubs or societies (ρ = 0.36).

It is interesting to note that the only significant correlations between student academic performance (measured by average grades) and levels of participation in learning activities are negative. Perhaps less surprising are negative correlations between grades and participation in “social” activities: using retail facilities (ρ = -0.22); and using catering facilities (ρ = -0.32). It is hard to explain the negative correlations between grades and attending a teaching session (ρ = -0.17). We return to this topic in the Discussion.

The wellbeing scales (effort and happiness) are positively correlated with each other (ρ = 0.30): students who put in more effort report greater happiness. Effort is positively correlated with several non-compulsory learning activities: using the VLE (ρ = 0.27); going to the library (ρ = 0.31); using career services (ρ = 0.30); using social media for learning (ρ = 0.36); and using the Internet for learning (ρ = 0.50). Effort is also positively correlated with using retail facilities (ρ = 0.27), perhaps suggesting more time spent on campus. Happiness is uncorrelated with core learning activities but is positively correlated with more social activities: using SU facilities (ρ = 0.28); and going to clubs or societies (ρ = 0.36).

Table 3 shows many positive correlations between levels of participation in various learning activities. Without listing all the pairwise relationships here, we find that 50% of activity pairs are significantly positively correlated, with no activity pairs negatively correlated. This suggests that students who engage more with learning do so in a holistic manner, with raised participation across a variety of learning activities.

### Temporal trends and correlations

**Fig 4:**
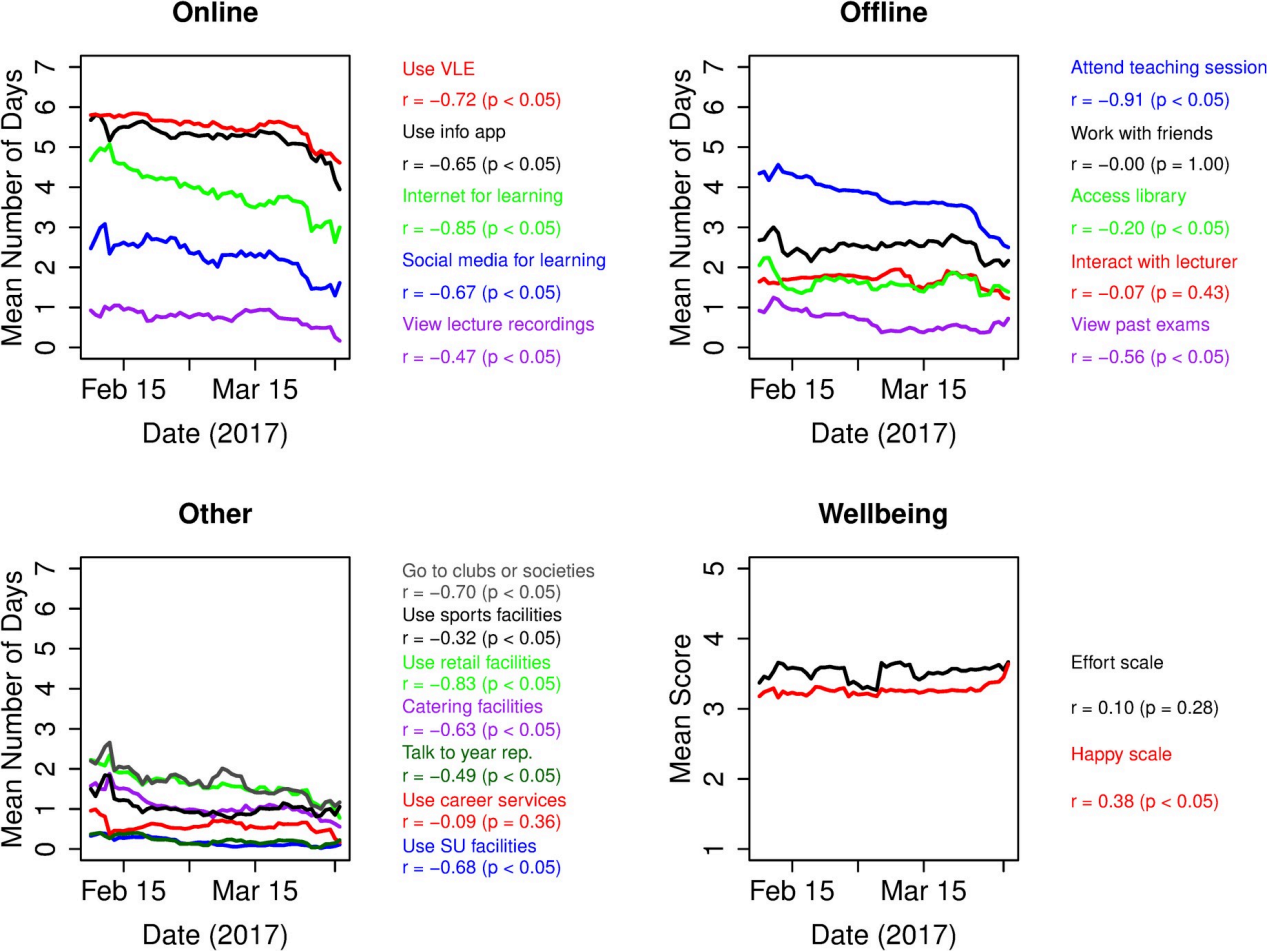
Trends in dynamic variables (engagement and wellbeing scores) over the term. Time series are calculated as a moving average using data from all students. Trends and significance are calculated using Kendall’s tau correlation coefficient.

Next, we consider trends or changes in behaviour during the Spring term (Fig 4), looking first at time series of reported participation levels for each learning activity (see Methods). Since we use a moving average to give robust values, and since survey response rate falls outside term time, we restrict our analysis to the period within the Spring term (Waves 1–7, prior to the grey shaded area in Fig 1). We use a moving average equal to one week (7 days) and when doing this, the lowest number of responses in any window is 17 (on the last day of term), suggesting the plotted values are reliable. Apart from the final two days of term, all the windows have 38 or more responses within them. Trends are calculated using Kendall’s tau correlation coefficient (see Methods). For ease of viewing, we have split the learning activities into ‘Online’ learning activities (Fig 4a), ‘Offline’ learning activities (Fig 4b) and ‘Other’ activities (Fig 4c). We also plot time series for wellbeing variables (Fig 4d).

There is a general downward trend in participation with learning activities over the Spring term. Of the ‘Online’ systems (Fig 4a), all of them have a significantly downward trend as the term goes on: using the VLE (τ = -0.72); using the info app (τ = -0.65); using the Internet for learning (τ = -0.85); using social media for learning (τ = -0.67); and accessing lecture recordings (τ = -0.47). Three of the ‘Offline’ systems also decrease over the term (Fig 4b): attending teaching sessions (τ = -0.91); accessing the library (τ = -0.20); viewing past exams (τ = -0.56). Since teaching activities are scheduled with a roughly uniform density throughout the term, the downward trend in engagement with learning activities is notable. A similar trend is seen for many of the ‘Other’ activities (Fig 4c): going to clubs or societies (τ = -0.70); using the sports facilities (τ = -0.32); using retail facilities (τ = -0.83); using catering facilities (τ = -0.63); talking to a year rep (τ = -0.49); using SU facilities (τ = -0.68). There are no learning activities that show an increase in participation over the term.

Looking at trends in the wellbeing variables over the term, we see that effort increases slightly but not significantly (τ = 0.10). However, happiness increases significantly (τ = 0.36), suggesting that students report greater happiness as the term progresses. We cannot say whether this increase in self-reported happiness is related to the concurrent decrease in engagement, though it is tempting to speculate.

Table 4 shows correlations between the dynamic variables measuring participation in learning activities and wellbeing. This analysis shows whether there are temporal associations between levels of participation in different activities (e.g., if a student does more of one activity, does this correspond to more engagement in other activities). The striking observation in this analysis is that nearly all pairwise relationships between dynamic variables show significant positive correlations, with a small number of exceptions. This indicates a pattern whereby student learning activity varies holistically; students may be more or less active, but when they are active, they are active across a wide range of activities and behaviours. Again, the two wellbeing scales are correlated with each other (ρ = 0.37). Overall, 83% of the pairwise relationships between learning activities show a positive correlation over time (compared to 50% for the averaged data shown in Table 3). We find two significant negative correlations: between viewing past exam papers and visiting a lecturer (ρ = -0.08) and attending a teaching session (ρ = -0.13). This is most likely because Table 4 uses time-resolved information and is affected by the switch between attendance at scheduled teaching sessions during the Spring term and using past exams to revise for upcoming exams during the Summer term.

**Table 4:** Dynamic correlations in the survey.

	2	3	4	5	6	7	8	9	10	11	12	13	14	15	16	17	18	19*
**1. Effort Scale**	0.37*	0.28*	0.15*	0.22*	0.35*	**-0.02**	0.29*	**0.02**	**-0.05**	**-0.02**	0.13*	0.10*	0.16*	0.44*	0.20*	**-0.10#**	0.09*	**-0.03**
**2. Happy Scale**		0.16*	0.14*	0.14*	0.16*	0.11*	0.11*	0.08*	**-0.05**	**0.01**	0.13*	0.13*	0.24*	0.22*	**-0.01**	**0.05**	0.07*	**-0.02**
**3. Friends**			0.24*	0.07*	0.23*	0.20*	0.25*	0.18*	**0.00**	0.14*	0.42*	0.34*	0.35*	0.32*	0.18*	0.13*	0.14*	**0.06**
**4. Lecturer**				0.17*	0.12*	0.27*	0.32*	**0.02**	0.08*	0.19*	0.25*	0.16*	0.16*	0.21*	**-0.08#**	0.10*	0.18*	**0.00**
**5. Info App**					0.54*	0.33*	0.24*	**-0.01**	0.11*	**0.02**	0.13*	0.11*	0.17*	0.24*	0.10*	0.08*	**0.05**	0.15*
**6. VLE**						0.22*	0.30*	0.08*	0.06*	**0.02**	0.12*	0.09*	0.20*	0.46*	0.20*	**0.05**	0.11*	0.10*
**7. Session**							0.10*	0.23*	0.09*	0.09*	0.22*	0.19*	0.17*	0.12*	**-0.13#**	0.40*	0.10*	0.15*
**8. Library**								0.17*	0.17*	0.17*	0.28*	0.24*	0.23*	0.35*	0.12*	0.13*	0.08*	**0.03**
**9. Sports**									**0.02**	**0.05**	0.16*	0.16*	0.07*	**0.04**	0.13*	0.39*	0.09*	0.08*
**10. Career**										0.20*	0.07*	0.11*	0.11*	**0.01**	**-0.00**	0.18*	0.18*	**0.03**
**11. Guild**											0.23*	0.25*	0.23*	0.08*	0.11*	0.24*	0.17*	0.16*
**12. SU**												0.58*	0.22*	0.22*	0.06*	0.24*	0.13*	0.12*
**13. Catering**													0.27*	0.20*	**0.06**	0.20*	0.15*	0.07*
**14. Soc. Med**														0.37*	0.10*	0.18*	0.15*	**0.00**
**15. Internet**															0.23*	**0.02**	0.14*	**0.00**
**16. P. Exams**																**0.01**	0.09*	0.21*
**17. Socs**																	0.14*	0.11*
**18. Year Rep**																		0.09*

Column 19 refers to ‘Lecture Recordings’.

Red boxes (*) refer to significantly (*p* < 0.05) positive correlations and blue (#) to significantly negative.

### Impact of assessments on engagement and wellbeing

**Fig 5:**
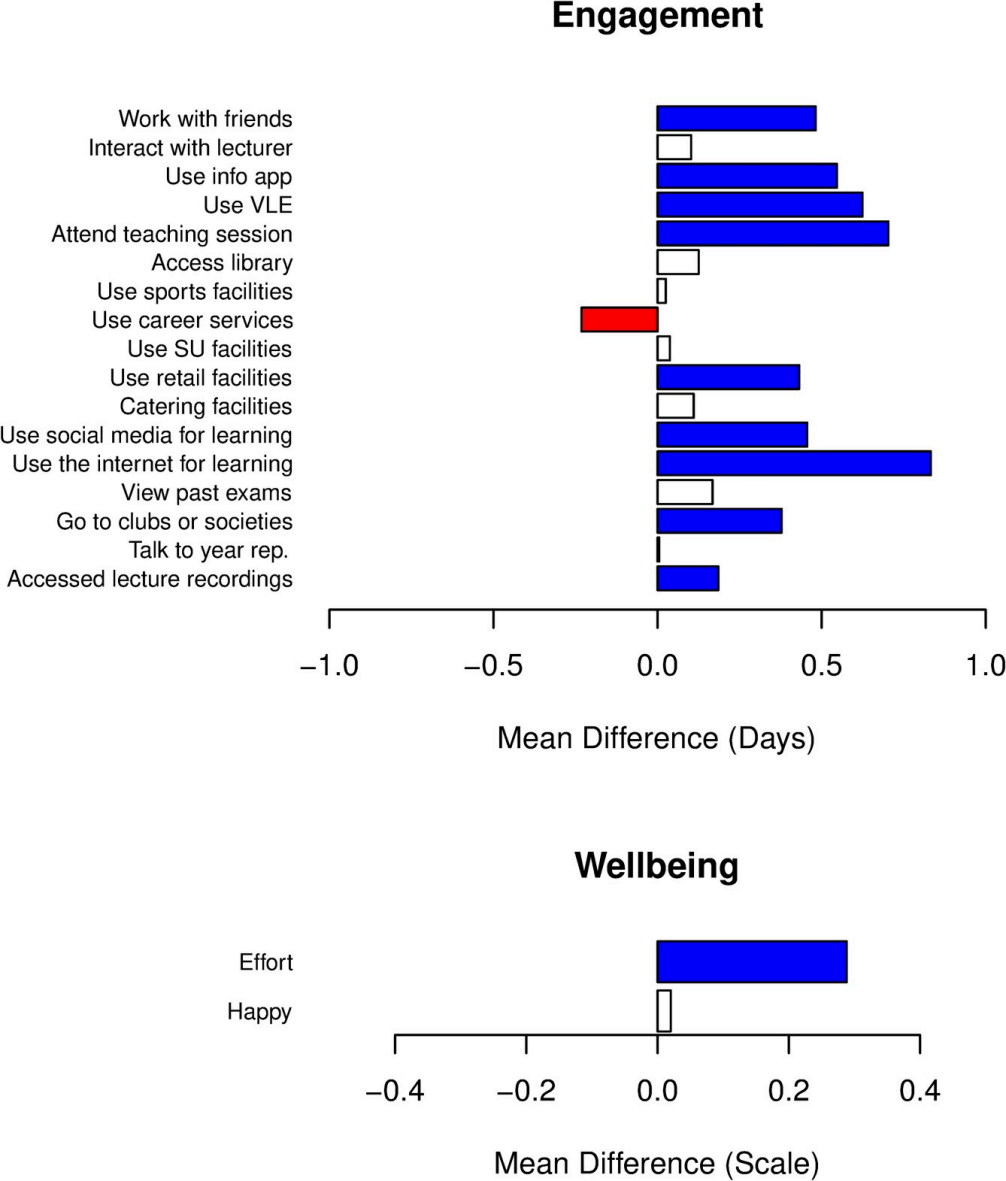
Differences in (upper panel) participation levels for different learning activities and (lower panel) wellbeing for responses from assessment and non-assessment weeks. Bars show the difference in mean values for reported score distributions for (upper panel) participation in each learning activity measured in days, or (lower panel) levels of effort and happiness on scale 1–5. Positive values indicate an increase in assessment weeks. Bar colours indicate statistical significance for the difference between distributions calculated from a Mann-Whitney U-test (blue—significant positive difference, red—significant negative difference, white—not significant).

To determine the impact of assessments (e.g., coursework, class tests, final exams, etc.) on student engagement and wellbeing, we split our dataset into “assessment week” responses (those responses where the student answered that there was an assessment due in the 7-day reporting period) and “non-assessment week” responses (where no assessments were due). Note that “assessment weeks” are temporally heterogeneous and specific to the individual; that is, the assessment/non-assessment weeks are not temporally correlated across the cohort. This rules out effects from globally correlated hidden variables such as, for example, campus wide events, external media stories, etc. For each set of responses, we create distributions for each dynamic variable and then measure the differences between these distributions using the difference in means and Mann-Whitney U-tests (see Methods). Results are shown in Fig 5. The bars in Fig 5 plot the difference in mean values for each distribution, with positive differences referring to increased participation in assessment weeks. Bar colours indicate whether the difference between the distributions is statistically significant according to the Mann-Whitney U-test.

Fig 5 (upper panel) shows the mean difference for assessment weeks and non-assessment weeks in the reported number of days of participation in each learning activity. We find increased participation in all learning activities during assessment weeks, except using career services, which had significantly less usage when an assessment was due. Of the activities with increased participation, 9 of the 15 increases were significant. Interestingly, increased participation in assessment weeks extends across a mix of activity types; for example, there is greater attendance at clubs and societies when assessments are due. Overall, the analysis suggests there is higher engagement with most learning activities when assessments are due.

We also look for differences in the wellbeing variables of effort and happiness between assessment weeks and non-assessment weeks (Fig 5, lower panel). We find that there is a significant increase in the effort levels students report when an assessment is due. There is also, perhaps surprisingly, a slight increase in happiness, although this is not significant.

### Relationships between behaviour and wellbeing

**Fig 6:**
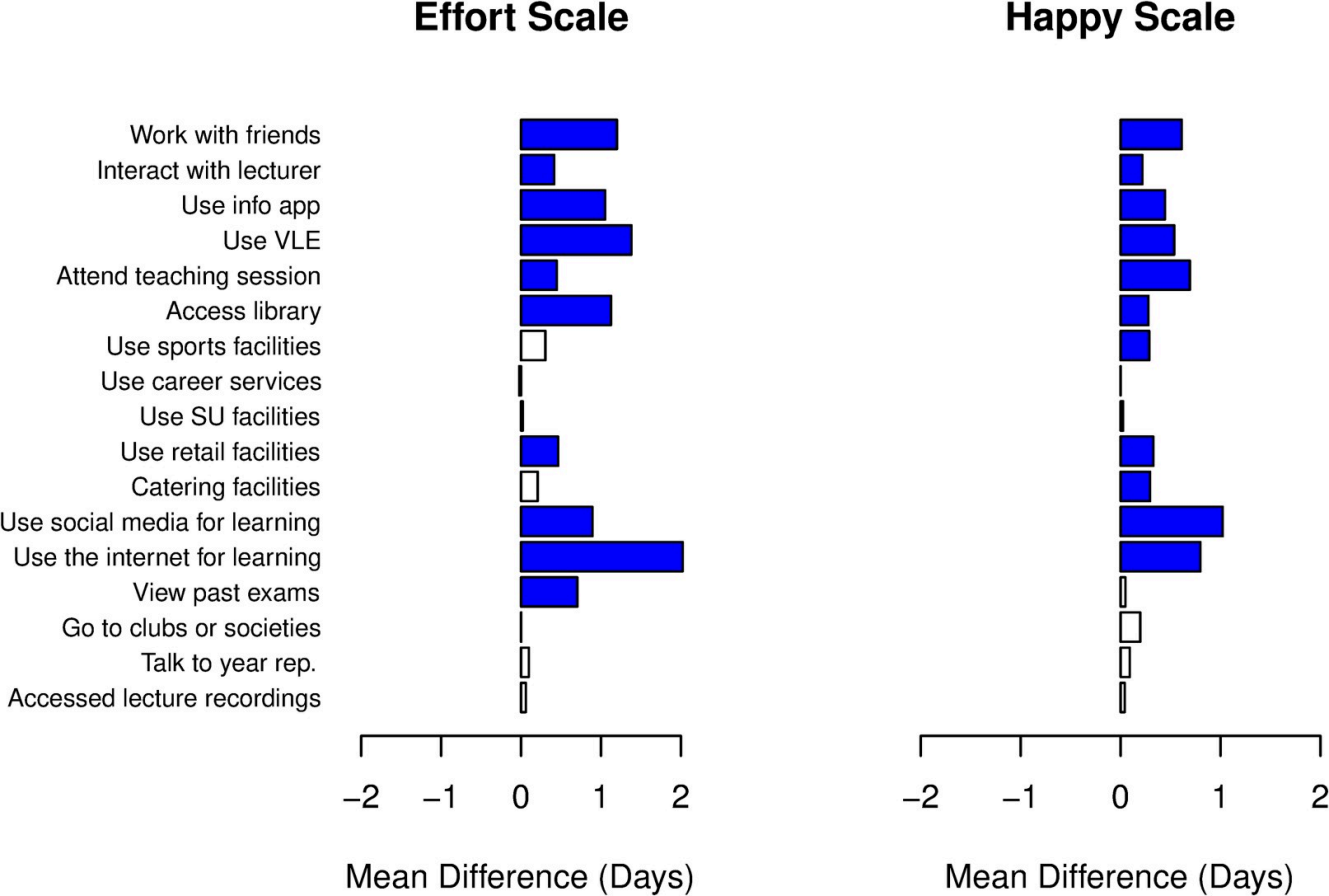
Differences in participation levels for (left panel) high effort students vs low effort students, and (right panel) happy students vs unhappy students. Bars show the difference in mean scores (in days) from the distributions of participation levels for different learning activities. Positive values indicate higher participation by the (left) high effort and right (happy) students. Bar colour indicates significant differences between the distributions according to a Mann-Whitney U-test (blue—significant positive difference, red—significant negative difference, white—not significant).

To explore the relationship between engagement with learning activities and reported wellbeing, we again split our dataset, this time into sets of responses where the student reported high/low levels of effort and high/low levels of happiness for that week. Since both variables are measured on an integer scale from 1 (low) to 5 (high), we use a threshold of 3 to split the cohort in each case, creating datasets for those who responded below 3 and those who reported 3 or above. This gives comparator sets for students who report “high effort” or “low effort” and students who report “happy” or “not happy”. Results are shown in Fig 6.

As expected, we find that 16 of the 17 learning activities show higher mean participation levels by high effort students, and for 10 of these the difference between the distributions is significant (Fig 6, left panel). Happy students have higher mean participation levels in all activities that students who are not happy (Fig 6, right panel). However, these differences are generally smaller than those for high vs low effort groups. When comparing the left and right panels in Fig 6, there is a significant increase in going to the Sports Park and using catering facilities for happier students, whereas rates of viewing past exams are only significantly increased for high effort students.

## Discussion

In planning this research, we expected to find different patterns of engagement among students, such as individuals showing more engagement with certain systems and less with others. This might be driven by students’ personal preferences (e.g., [27, 28]) or by the teaching activities prescribed and/or preferred by different disciplines and programmes (see e.g. [8, 41]). Instead we find that students who are engaged with learning tend to be engaged with all learning activities and systems; engagement appears to be a holistic phenomenon (Tables 3 and 4). The only exception to this pattern is a negative correlation between attending scheduled teaching sessions and viewing past exam papers. This might be explained by the separation (for most students) of learning and revision, with exam papers used for revision after scheduled teaching has finished. The strong correlation between all forms of engagement with learning has possible instrumental value for the design of systems to monitor student engagement, since it suggests that engagement could be effectively tracked using only a subset of engagement metrics as indicators. Monitoring of engagement might be used to identify anomalies or changes in behaviour of individuals, for example, to assist tutors in providing support and pastoral care. Indeed, the predictive analytics project at Nottingham Trent University (NTU Student Dashboard), which calculates engagement scores based on five online resources (VLE access, library usage, attendance, assignment submissions, and card swipes), has identified a positive relationship between student engagement and both progression and attainment. Moreover, this information, when communicated to students and staff, has been used to provide more targeted support to students from pastoral tutors (see [42]).

A feature of our survey design is the ability to measure variables at a campus-based university that would otherwise be difficult to access. Of the 17 learning activities recorded by our survey, only four could be tracked digitally with current methods (VLE, info app, past exam views and recorded lecture viewing), with the rest not routinely measured. Furthermore, this study provides temporally resolved data on student wellbeing, giving the opportunity to explore relationships between engagement and wellbeing.

Engagement and wellbeing are shown in this study to be positively related. Looking longitudinally across the survey (Table 4), we find 13 forms of engagement were positively (and significantly) correlated with at least one of the wellbeing variables, either effort or happiness. Reasonably, one could suggest a possible feedback loop where increasing engagement increases academic performance, which in turn increases wellbeing (happiness and grades are correlated; Table 4), which then increases engagement. Alternatively, students with greater background levels of wellbeing may be more likely to engage with learning (see also [30, 31]). This study cannot separate these potential mechanisms, since it only shows correlation and cannot assign causality.

The responses to our survey show a broad sample of student engagement at the university where the study was based. The survey was widely advertised and contains responses from students across all disciplines. However, in common with most survey studies, it relies on voluntary participation and we had no control over who would participate (see also [43]). This may introduce bias into our results. For example, we find that the students who responded scored much higher on academic motivation than on social motivation (Fig 2), but this may be an artefact of self-selection bias in the sample of survey respondents, such that academically motivated students who are engaged with learning were more likely to participate (see also [43, 44]). Indeed, analysis of the demographic data of respondents suggests that certain disciplines were over-sampled. This might limit the generalizability of our findings to the whole cohort, given that there are likely to be disciplinary differences in the extent to which students are expected to engage with various learning systems (see [8]). Furthermore, since this study was based at a single university in the UK, it may not represent students at other universities in the UK or worldwide. We encourage other researchers to repeat our study at other institutions in order to consolidate our findings. We make our survey design available in the Supplementary Information (S1 File) to facilitate this.

Another caveat to our results is that differences between student workloads associated with different learning activities are not considered. In previous work, we have shown that the amount of observed VLE usage differs between different disciplines [8], explained by the differing requirements of different disciplines, programmes and modules. For example, a humanities student is likely to have a balance of learning activities that differs from an engineering student, with resulting variation in the time they spend on the VLE. In addition, the number of scheduled lectures and other contact hours will differ between disciplines, with students taking STEM subjects typically having more contact hours than those taking arts or humanities subjects which require more self-study. It is possible that these differences might affect some of our findings. For example, the correlation between attending scheduled teaching sessions and student happiness might be influenced by the fraction of sessions attended, rather than the absolute number; a student who attends 100% of 4 scheduled sessions might be happier than a student who attends 50% of 8 scheduled sessions, even though the number of attended sessions remains the same. This kind of difference might mask or confound some relationships, so it is possible that a study sample stratified on discipline or programme would give a more nuanced picture of the relationships between engagement and wellbeing. With a larger sample size, we would have been able to create disciplinary subsets of students to explore this aspect, but our sample size did not permit this here.

One interesting dimension of student engagement that we are yet to explore within our survey is how well students predict their own usage of various learning systems; that is, do they accurately report their usage of digital tools? Results given here are based on student self-report rather than documented usage of different systems. In general, students might mis-report their behaviour either by mistake or deliberately, for whatever reason. If self-reported data in the current survey are inaccurate, it might raise the interesting question of whether some students systematically under- or over-report their levels of engagement with learning, and whether students who misreport perform better or worse academically (see [45, 46]). We will return to this question in future work. If self-report and documented data (where available) do not agree, it raises the question of which sources show a more accurate picture of student behaviour and which are more important in relation to student wellbeing.

We can only speculate why there is an observed decrease in engagement during the academic term. It could be because students like to get ahead at the start of term and work harder or engage more to do this. The larger drop off in engagement at the end of term may be due to students having assessments that are not due until after the break and therefore not needing to work as much as they do during the middle of term. The rise in reported effort during the term (although not statistically significant) is interesting in relation to the decrease in reported engagement. The observed increase in happiness towards the end of term seems to be robust but is hard to explain; we speculate that perhaps students become happier as they start to receive assessment outcomes, or maybe they are simply looking forward to the end of term. This may be at odds with the correlations between engagement and wellbeing discussed previously. However, we believe that the correlations are picking out individual student behaviours, whereas these trends reflect the whole population.

Our research identified strong differences in behaviour between students who have an assessment due and those who do not. This gives us confidence that our survey can identify meaningful results, despite the limited sample size. We also find strong differences in behaviour between those students who feel engaged as well as happy. Finding that students who are happy are engaging more is an important result for our understanding of student wellbeing. Coupled with mechanisms to routinely measure engagement, it could assist tutors to identify students who are suffering with poor wellbeing and might benefit from intervention or greater support.

## Supporting information

S1 File Questions used in survey completed by participants. The original survey was completed using survey software Qualtrics.
(PDF)
