# Supplementary material for: Student engagement and wellbeing over time at a higher education institution
Source: PLoS One. 2019 Nov 27;14(11):e0225770. doi: 10.1371/journal.pone.0225770 (PMC6881016; doi:10.1371/journal.pone.0225770)
Supplement: S1 File — The original survey was completed using survey software Qualtrics. (PDF) [file pone.0225770.s001.pdf]

INFORMATION SHEET/CONSENT FORM

What we are doing
As part of the University of Exeter's Effective Learning Analytics project, we are collecting data from students about their engagement with different resources and facilities at the University of Exeter. We will use the data provided by students in this research alongside digital data available about students' usage patterns to develop a greater understanding of how students' behaviour is associated with achievement and retention.

What we are asking you to do
If you agree to take part, you will be asked to complete a short survey about your behaviour each week in Term 2 – 11 surveys in total. You will be emailed a link to the survey each week. The questions ask you about your use of different resources/facilities available to students each week.

You will also be asked to give consent for us to match the responses you give us to our questions with data recorded by the University of Exeter, such as your grades. For example, you will be asked about how often you have used ELE and then we would match this with your usage recorded by the ELE system.

Each weekly survey should only take about 5 minutes to complete. The first survey should take about 15 minutes to complete as it includes questions that we'll only ask you once.

What happens to the information that you give us
You do not have to take part in the survey if you don't want to. But by completing it, you will help us to better understand the factors that are associated with student performance.

Any information that you give us will be treated in strict confidence. Only the researchers will know that you have completed the questionnaire. Any personally identifying information will be removed from the datasets before we conduct our analyses: you will not be identified by anything you say.

What are the benefits for you?
In addition to helping us advance knowledge in this field, **each week** participating students will be entered into **a prize draw to win one of 10 £20 Amazon vouchers**. In addition, students who complete **8 or more surveys** will be entered into a final prize draw to **win one of 10 £50 Amazon vouchers**.

Ethical approval
This research has been approved by the Psychology Ethics Committee in line with the guidelines of the British Psychological Society.

Right to withdraw
If you do not wish to answer a particular question on the survey, you do not have to. Any information that you give us is valuable. If you change your mind about participating at any time over the course of Term 2, you can do so by contacting the Project Contact person below.

Project contact

<u>Consent Form</u>

To sign up to participate in this research, please read through the statements below and continue with the study if you agree with each statement.

1. I have had the opportunity to consider the information about this study.

2. I understand that my participation in this study is voluntary and that I can withdraw from the study at any time.

3. I understand that by agreeing to participate, I give consent for my survey responses to be matched, based on my student number, with other data held by the University of Exeter about me.

4. I understand that my data will be confidential and anonymised prior to analysis by the research team.

5. I know how to contact the research team if I need to.

QUESTIONS TO BE ASKED EACH WEEK

First, we'd like to ask you some questions about your engagement with a number of resources, facilities, and activities at the University of Exeter. Please indicate on how many days over the past week you accessed each resource.

Over the last week, on how many days did you:

Work/study with friends
Interact with a lecturer
Use iExeter
Use ELE
Attend a scheduled teaching session (e.g., lecture)
Access library resources
Use the Sports Park (e.g., gym, fitness classes)
Access the Career Zone
Use Guild facilities (e.g., Voice, advice services)
Use the retail facilities on campus (e.g., Marketplace)
Use non-university social media platforms (e.g., Facebook, Twitter) for learning
Use the internet for learning (e.g., to find papers, tutorials etc)
Access past exam papers
Participate in clubs and societies

0/7 days	1/7 days	2/7 days	3/7 days	4/7 days	5/7 days	6/7 days	7/7 days

Now, we'd like to ask you about how you felt about your studies over the last week.

Over the last week:

How engaged were you with your studies?
How satisfied were you with the quality of your course?
How much effort did you put into your studies?
How much did you feel part of a community of staff and students?
How happy did you feel about your life in general?
How well do you feel you are doing in your course?

1	2	3	4	5
Not at all				Very much

Did you get a result back this week?
Yes/no

If yes, how satisfied were you with the mark you received?

1	2	3	4	5
Not at all				Very much

Did you have an assessment due this week?

Yes/no

If yes, how much effort did you put into the assessment?

1	2	3	4	5
Not very much				A lot

ADDITIONAL QUESTIONS FOR TIME 1

STUDENT ENGAGEMENT

In this next section, we're interested in how you feel about your learning experience at university. Please answer by indicating your level of agreement with each statement. [1 strongly disagree, to 5 strongly agree]

[behavioural engagement]

I try hard to do well at university

In my modules, I work as hard as I can

When I'm in class, I participate in class discussions

When I'm in class, I just act like I'm working

In my modules, I do just enough to get by

If I have trouble understanding something, I go over it again until I understand it

[cognitive engagement]

When I study, I try to understand the material better by relating it to things I already know

When I study, I figure out how the information might be useful in the real world

When I study, I try to combine different pieces of information from my modules in new ways

When I study, I try to connect what I am learning with my own experiences

LEARNING STYLES

Below are 12 sentences with a choice of four endings. Rank the endings for each sentence according to how well you think each one fits with how you would go about learning something, from 4 *most descriptive of me* to 1 *least descriptive of me*. Try to recall some recent situations where you had to learn something new, perhaps in your job. In ranking the words, use your first impression. There are no right or wrong answers.

**1. When I learn...**	...I like to deal with my feelings ________	...I like to watch and listen ________	...I like to think about ideas ________	...I like to be doing things. ________
**2. I learn best when...**	...I trust my hunches and feelings ________	...I listen carefully and watch ________	...I rely on logical thinking ________	...I work hard to get things done ________
**3. When I am learning...**	...I have strong feelings and reactions ________	...I am quiet and reserved. ________	...I tend to reason things out ________	...I am responsible about things ________
**4. I learn by...**	...feeling ________	...watching ________	...thinking ________	...doing ________
**5. When I learn...**	...I am open to new experiences ________	...I look at all sides of an issue ________	...I like to analyse things, break them into their parts ________	...I like to try things out ________
**6. When I am learning...**	...I am an intuitive person ________	...I am an observant person ________	...I am a logical person ________	...I am an active person ________
**7. I learn best from ...**	...personal relationships ________	...observation ________	...rational theories ________	...a chance to try and practice ________
**8. When I learn...**	...I feel personally involved ________	...I take my time before acting ________	...I like ideas and theories ________	...I like to see results from my work ________
**9. I learn best when...**	...I rely on my feelings ________	...I rely on my observations ________	...I rely on my ideas ________	...I can try things out for myself ________
**10. When I am learning...**	...I am an accepting person ________	...I am a reserved person ________	...I am a rational person ________	...I am a responsible person ________
**11. When I learn ...**	...I get involved ________	...I like to observe ________	...I evaluate things ________	...I like to be active ________
**12. I learn best when...**	...I am receptive and open-minded ________	...I am careful ________	...I analyse ideas ________	...I am practical ________
**Total Score**	________________	________________	________________	________________

MOTIVATIONS FOR STUDY

People choose to study at university for many reasons. Please indicate the extent to which each reason motivates you to study at the University of Exeter. (1 not at all, 5 very much)

To get a good job
To increase my knowledge and understanding
To please my family
To socialise with my friends

To continue on with my non-academic interests (e.g., playing sport)
To get good grades

OTHER IT SYSTEMS USED IN LEARNING
To what extent do you use the following IT systems/social media platforms while studying at the University of Exeter? (1 not at all, 5 all the time)

Facebook
Twitter
Google products (e.g., docs, Scholar)
